# Pasteur’s Laboratory

**Published:** 1886-12

**Authors:** 


### PASTEUR'S LABORATORY.

The Pasteur pavilion is a detached house one story high, standing at right angles to the main building of the Ecole Normale, and facing the broad gravelled stretch between the front of the institution and the Rue d' Ulm. In its facade there is a central hall door, flanked on each side by a couple of windows. The two nearest to it light the hall, a square room papered in dark green and wainscoted in pale oak. To the right there is a parlor, which is also a good deal of a chemist's workshop, and to the left a study with two windows, one of which is on the north and the other on the west side of the room. Everything has a workshop air. The book cases are not glazed. The portfolios on the shelf are of the commonest sort. On other shelves there are retorts, tubes, phials and microscopes. The tables are deal, painted black. A man sits with his face to a window, in the west or hall door facade, in the early hours of the forenoon. From nine until half-past ten he is to be seen opening, reading and sorting telegrams and cablegrams. He files some and flings others into a large basket near him, which is full to overflowing before the inoculations begin. As his back is to anyone entering the room, to see his face it is necessary to return to the gravelled space and look at him with the full light of day pouring in on his face, quite unconcerned at the two hundred and fifty pairs of eyes that are gazing at him. Pasteur may be clerical or reactionary, but he is *egalitaire* when he treats for rabies, and pays no heed to external signs of rank and wealth. He is obliging to all in the manner of a kindly, hardworked man, who has no time to lose in idle talk or empty compliments. His conversation with a newcomer, however important or well introduced, is limited to "How do you do? What can I do for you?" This not dryly or gruffly. And on being told that the visitor wants to be inoculated, he says, "Good. Go and wait your turn with the others." He asks very few questions, indeed sometimes none, about how applicants for treatment came to be bitten, and does not like to hear that the dog which inflicted the bite has not been killed.—*Medical Summary.*

